# Crystal structures of three *N*-(aryl­sulfon­yl)-4-fluoro­benzamides

**DOI:** 10.1107/S2056989016005089

**Published:** 2016-03-31

**Authors:** P. A. Suchetan, S. Naveen, N. K. Lokanath, K. S. Srivishnu, G. M. Supriya, H. N. Lakshmikantha

**Affiliations:** a Department of Chemistry, University College of Science, Tumkur University, Tumkur 572 103, India; b Institution of Excellence, University of Mysore, Manasagangotri, Mysuru-6, India; c Department of Studies in Physics, University of Mysore, Manasagangotri, Mysuru-6, India; d University College of Science, Tumkur, India

**Keywords:** crystal structure, *N*-(aryl­sulfon­yl)aryl­amides, N—H⋯O hydrogen bonds, O—H⋯O hydrogen bonds, C—H⋯O inter­actions

## Abstract

The crystal structures of three *N*-aryl­sulfonyl-4-fluoro­benzamides, namely 4-fluoro-*N*-(2-methyl­phenyl­sulfon­yl)benzamide, C_14_H_12_FNO_3_S, (I), *N*-(2-chloro­phenyl­sulfon­yl)-4-fluorobenzamide, C_13_H_9_ClFNO_3_S, (II), and *N*-(4-chloro­phenyl­sulfon­yl)-4-fluoro­benzamide monohydrate, C_13_H_9_ClFNO_3_S·H_2_O, (III), are described and compared with related structures. The asymmetric unit of (I) contains two independent mol­ecules (*A* and *B*), while that of (II) contains just one mol­ecule, and that of (III) contains a mol­ecule of water in addition to one main mol­ecule. The dihedral angle between the benzene rings is 82.83 (11)° in mol­ecule *A* and 85.01 (10)° in mol­ecule *B* of (I), compared to 89.91 (10)° in (II) and 81.82 (11)° in (III). The crystal structure of (I) features strong N—H⋯O hydrogen bonds between the *A* and *B* mol­ecules, resulting in an *R*_4_^4^(16) tetra­meric unit. These tetra­meric units are connected into sheets in the *bc* plane by various C—H⋯O inter­actions, and adjacent sheets are further inter­linked *via* C—H⋯π_ar­yl_ inter­actions, forming a three-dimensional architecture. The crystal structure is further stabilized by π_ar­yl_–π_ar­yl_ and S=O⋯π_ar­yl_ inter­actions. In the crystal of (II), mol­ecules are connected into *R*_2_^2^(8) and *R*_2_^2^(14) dimers *via* N—H⋯O hydrogen bonds and C—H⋯O inter­actions, respectively; the dimers are further inter­connected *via* a weak C=O⋯π_ar­yl_ inter­action, leading to the formation of chains along [1-10]. In the crystal of (III), N—H⋯O and O—H⋯O hydrogen bonds involving both the main mol­ecule and the solvent water mol­ecule results in the formation of sheets parallel to the *bc* plane. The sheets are further connected by C—H⋯O inter­actions and weak C—Cl⋯π_ar­yl_, C—F⋯π_ar­yl_ and S=O⋯π_ar­yl_ inter­actions, forming a three-dimensional architecture.

## Abstract 2

The crystal structures of three *N*-(aryl­sulfon­yl)-4-fluoro­benzamides, namely 4-fluoro-*N*-(2-methyl­phenyl­sulfon­yl)­benzamide, (I), *N*-(2-chloro­phenyl­sulfon­yl)-4-fluoro­benzamide, (II), and *N*-(4-chloro­phenyl­sulfon­yl)-4-fluoro­benzamide monohydrate, (III), are described and compared with related structures. The conformation of the three mol­ecules is very similar with the aromatic rings being inclined to one another by 82.83 (11) and 85.01 (10)° in the two independent mol­ecules of (I), 89.91 (10)° in (II) and 81.82 (11)° in (III).

## Chemical context   

Sulfonamide and amide moieties play a very significant role as key constituents in a number of biologically active mol­ecules (Mohan *et al.*, 2013 ▸; Manojkumar *et al.*, 2013 ▸; Hamad & Abed, 2014 ▸). In recent years, *N*-(aryl­sulfon­yl)aryl­amides have received much attention as they constitute an important class of drugs for Alzheimer’s disease (Hasegawa & Yamamoto, 2000 ▸), as well as antibacterial inhibitors of tRNA synthetases (Banwell *et al.*, 2000 ▸), antagonists for angiotensin II (Chang *et al.*, 1994 ▸) and leukotriene D4-receptors (Musser *et al.*, 1990 ▸). Further, *N*-(aryl­sulfon­yl)aryl­amides are known to be potent anti-tumour agents against a broad spectrum of human tumour xenografts (colon, lung, breast, ovary and prostate) in nude mice (Mader *et al.*, 2005 ▸). In view of the importance of *N*-(aryl­sulfon­yl)aryl­amides, the title compounds, (I), (II) and (III), were synthesized and we report herein on their crystal structures.
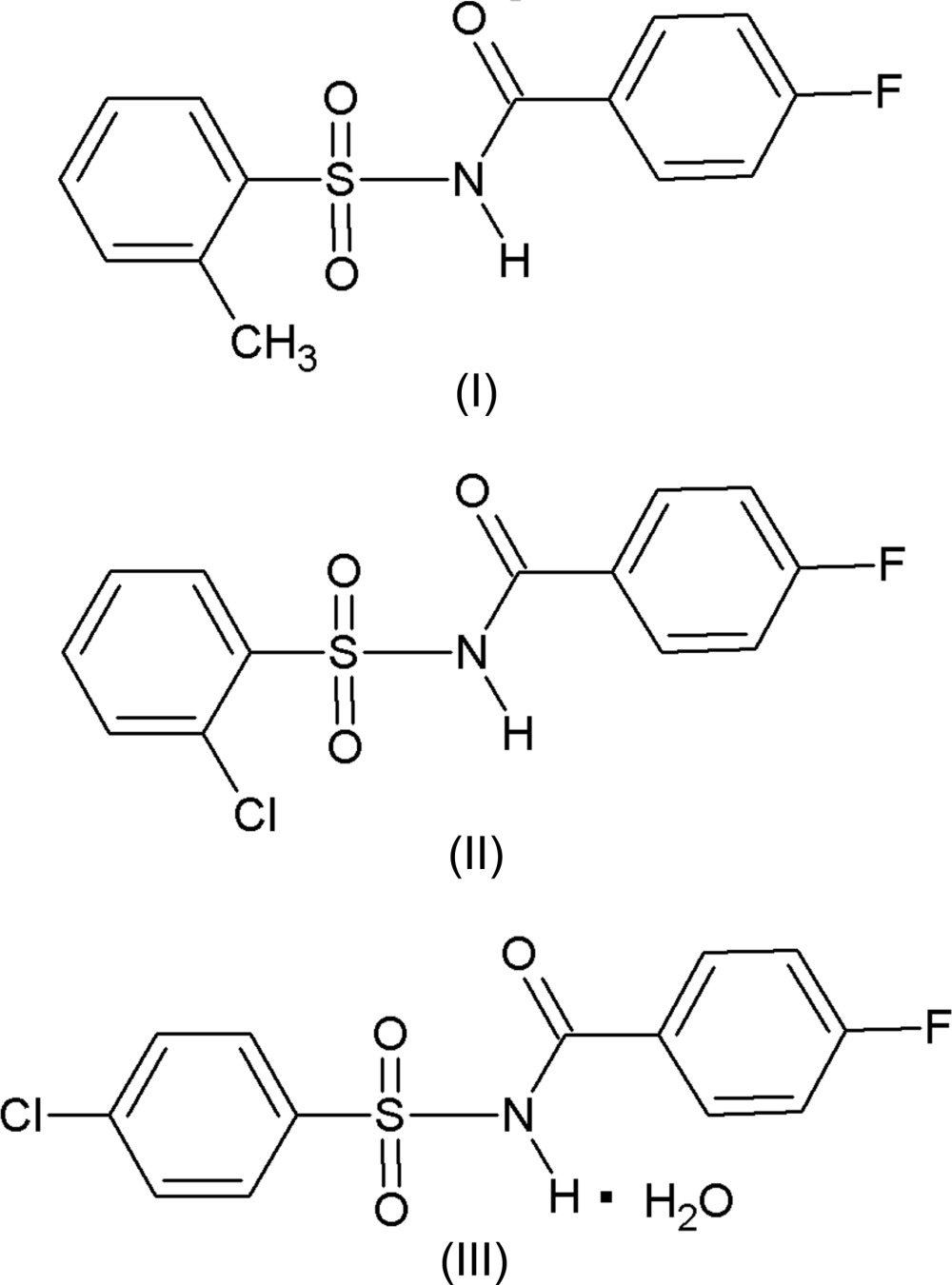


## Structural commentary   

**Figure 1:**
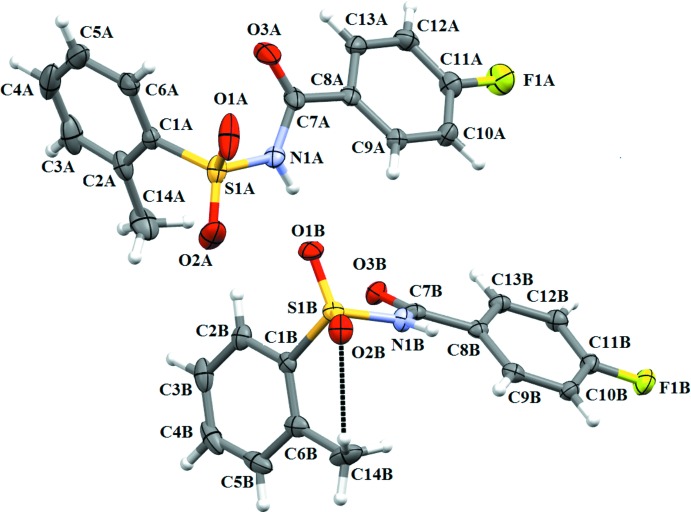
A view of the mol­ecular structure of the two independent mol­ecules (*A* and *B*) of compound (I), with atom labelling. Displacement ellipsoids are drawn at the 50% probability level.

**Figure 2:**
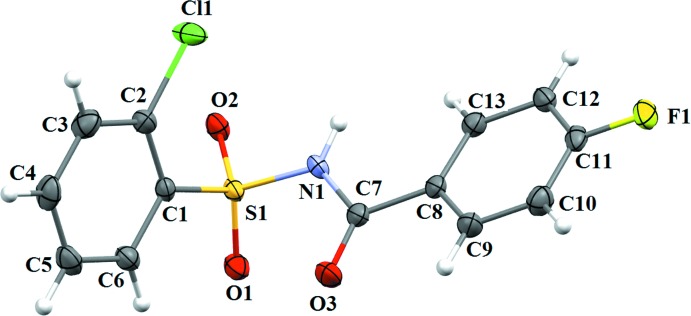
A view of the mol­ecular structure of compound (II), with atom labelling. Displacement ellipsoids are drawn at the 50% probability level.

**Figure 3:**
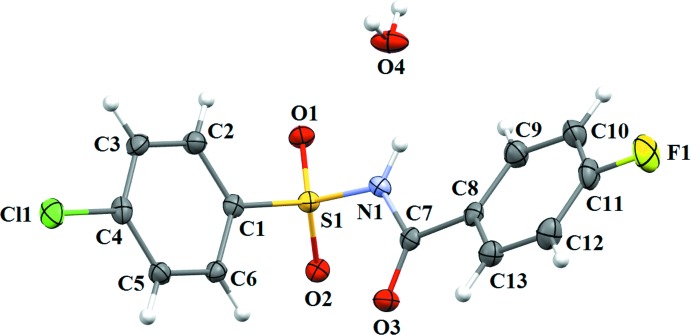
A view of the mol­ecular structure of compound (III), with atom labelling. Displacement ellipsoids are drawn at the 50% probability level.

The asymmetric unit of compound (I) contains two independent mol­ecules (*A* and *B*) (Fig. 1 ▸), that differ slightly in their mol­ecular conformations. The asymmetric unit of compound (II) (Fig. 2 ▸) contains one mol­ecule, while compound (III) (Fig. 3 ▸) crystallizes as a water monosolvate. In mol­ecules *A* and *B* of (I), the *ortho*-methyl substituent on the benzene­sulfonyl ring is *syn* to the N—H bond in the central –C–SO_2_–N–C(O)– segment (Fig. 1 ▸). This is similar to the *syn* conformation observed for the N—H bond in the central –C–SO_2_–N–C(O)– segment with respect to the *ortho*-chloro substitution on the benzene­sulfonyl ring of (II). The dihedral angle between the benzene rings is 82.83 (11)° in mol­ecule *A* and 85.01 (10)° in mol­ecule *B* of (I), compared to 89.91 (10)° in (II) and 81.82 (11)° in (III). Further, in (I) the dihedral angles between the benzoic acid ring and the central C8–C7(O3)–N1–S1 segment are 28.99 (1) and 23.81 (9)° in mol­ecules *A* and *B*, respectively, while it is 10.41 (10)° in (II) and 21.23 (10)° in (III). The dihedral angles between the sulfonamide ring and the C7(O3)–N1–S1–C1 segment are, respectively, 68.67 (12) and 77.31 (10)° in mol­ecules *A* and *B* of (I). The corresponding dihedral angle in (II) is 70.77 (11)°, whereas in (III) the value is much less, *viz* 48.03 (12)°. An intra­molecular C14*B–*-H14*B*⋯O2*B* hydrogen bond (Fig.1 and Table 1 ▸) is observed in mol­ecule *B* of (I), with an *S*(6) ring motif.

## Supra­molecular features   

**Figure 4:**
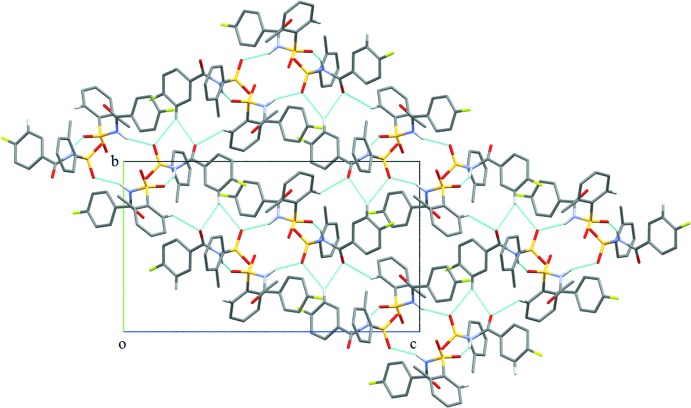
Crystal packing of (I), displaying N—H⋯O hydrogen bonds and C—H⋯O inter­molecular inter­actions (dashed lines), which result in the formation of sheets parallel to the *bc* plane.

**Figure 5:**
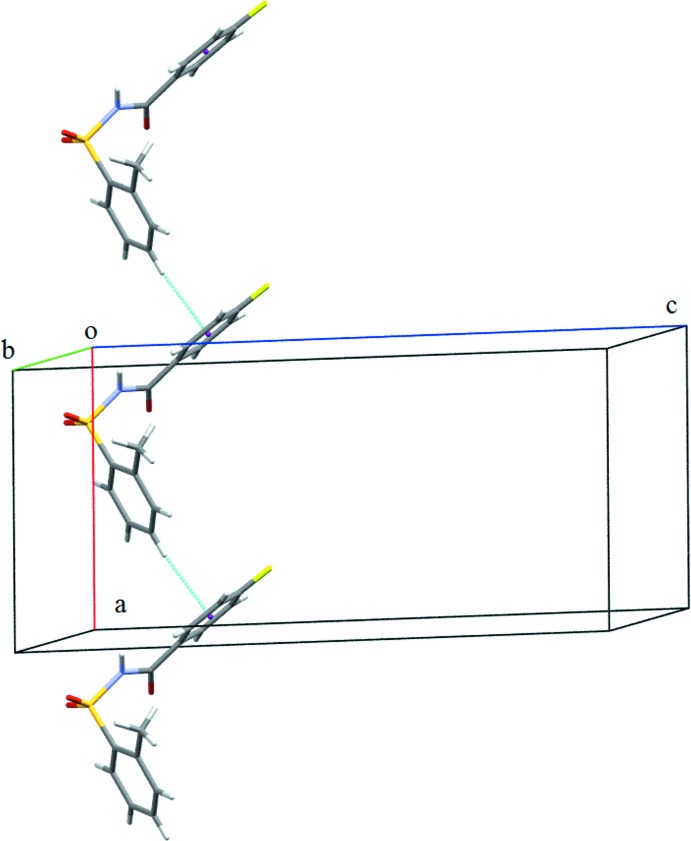
C—H⋯π_ar­yl_ inter­actions (dashed lines) displayed in (I).

**Figure 6:**
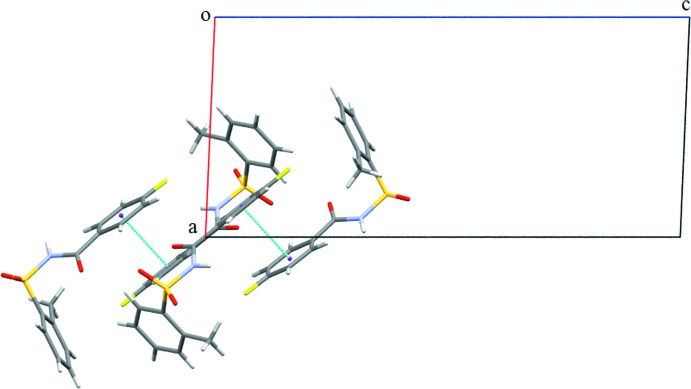
π–π inter­actions (dashed lines) displayed in (I).

The crystal structure of (I), features two strong N—H⋯O hydrogen bonds, namely, N1*A*—H1*A*⋯O1*B* and N1*B*—H1*B*⋯O1*A* hydrogen bonds (Table 1 ▸) between the *A* and *B* mol­ecules, resulting in a tetra­meric unit (Fig. 4 ▸). The unitary level graph-set notation for each hydrogen bond is *D*(2), while in the second level the tetra­meric unit has a graph-set motif of *R*_4_^4^(16). Adjacent tetra­mers are connected into sheets in the *bc* plane (Fig. 4 ▸) *via* C6*A*—H6*A*⋯O3*B*, C10*B*—H10*B*⋯O1*B* and C10*B*—H10*B*⋯O3*B* inter­actions (Table 1 ▸). Adjacent sheets are further inter­connected *via* C4*B*—H4*B*⋯π_ar­yl_ inter­actions (involving the centroid of the fluoro­benzoyl ring of mol­ecule *B*) (Fig. 5 ▸ and Table 1 ▸) to form chains along the *a* axis, so forming a three-dimensional architecture. The crystal structure of (I), is further stabilized by π_ar­yl_–π_ar­yl_ inter­actions (Fig. 6 ▸) [*Cg*1⋯*Cg*2 = 3.7413 (12) Å; *Cg*1 and *Cg*2 are the centroids of the fluoro­benzoyl rings of mol­ecules *A* and *B*, respectively] and also by weak S1*A*=O2*A*⋯π_ar­yl_ inter­actions [O⋯*Cg*3 = 3.7991 (19) Å; *Cg*3 is the centroid of the benzene­sulfonyl ring of mol­ecule *B*].

**Figure 7:**
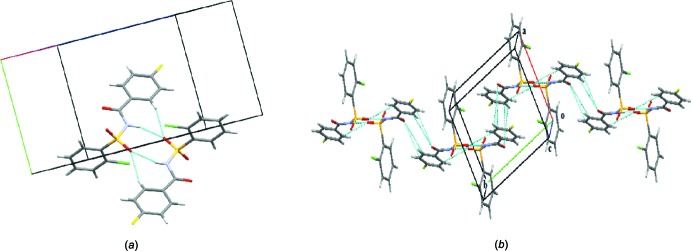
Crystal packing of (II): (*a*)display of 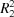
(8) and 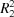
(14) dimers formed *via* N—H⋯O hydrogen bonds and C—H⋯O inter­actions, both shown as dashed lines; (*b*) formation of one-dimensional architecture.

In the crystal of (II), mol­ecules are connected into 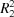
(8) dimers *via* N1—H1⋯O2 hydrogen bonds (Fig. 7 ▸ *a* and Table 2 ▸), and the dimers are further inter­connected *via* C13—H13⋯O2 inter­actions (Fig. 7 ▸ *a* and Table 2 ▸) with an 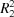
(14) graph-set motif. Weak C7=O3⋯π_ar­yl_ inter­actions [O⋯*Cg* = 3.9157 (19) Å; *Cg* is the centroid of the fluoro­benzoyl ring] connect these dimers, thus forming a one-dimensional architecture (Fig. 7 ▸ *b*).

**Figure 8:**
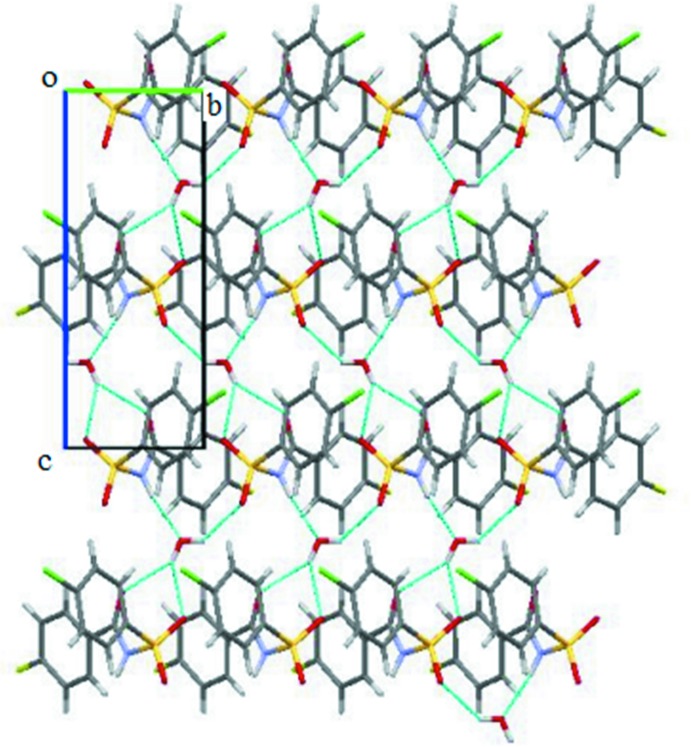
Crystal packing of (III), displaying an infinite two-dimensional sheet parallel to the *bc* plane formed *via* N—H⋯O and various O—H⋯O hydrogen bonds (dashed lines).

**Figure 10:**
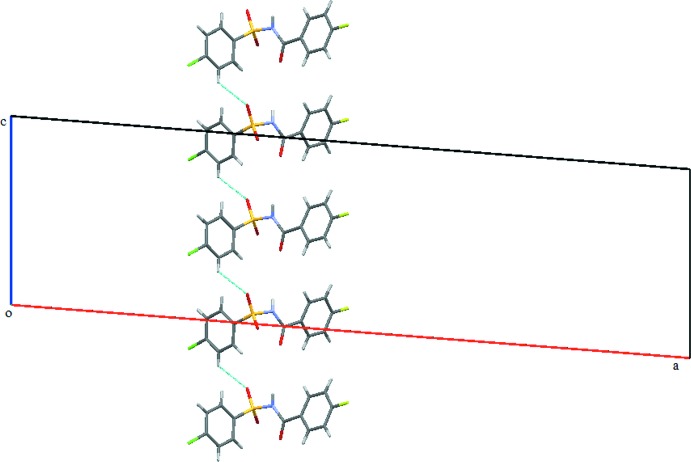
Display of C5—H5⋯O1 *C*(6) chains (dashed lines) running parallel to the *c* axis in (III).

**Figure 11:**
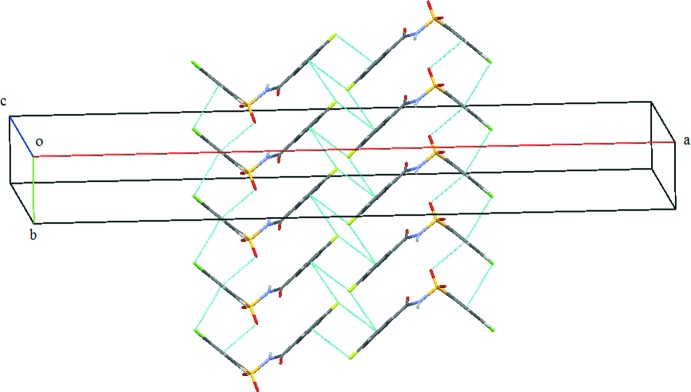
Display of various weak inter­actions (dashed lines) in the crystal structure of (III).

In the crystal of (III), mol­ecules are connected *via* bridging water mol­ecules, through strong N1—H1⋯O4, O4—H1*O*4⋯O1, O4—H2*O*4⋯O2 and O4—H2*O*4⋯O3 hydrogen bonds (Table 3 ▸), resulting in the formation of sheets parallel to the *bc* plane (Figs. 8 ▸ and 9 ▸). The sheets are further connected by C5—H5⋯O1 inter­actions, forming *C*6 chains (Table 3 ▸) running parallel to the *c* axis (Fig. 10 ▸). The crystal structure is also stabilized by several weak C—H⋯π inter­actions, C4—Cl1⋯*Cg*1 [Cl⋯*Cg*1 = 3.7513 (11) Å], C11—F1⋯*Cg*2 [F1⋯*Cg*2 = 3.8674 (17) Å] and S1=O2⋯*Cg*1 inter­actions [O2⋯*Cg*1 = 3.2039 (17) Å] (*Cg*1 and *Cg*2 are the centroids of the benzene­sulfonyl ring and fluoro­benzoyl rings, respectively), forming a complex three-dimensional architecture (Fig. 11 ▸).

## Database survey   

A search of the Cambridge Structural Database (CSD, Version 5.37, last update February 2016; Groom & Allen, 2014 ▸) for similar compounds *viz N*-(aryl­sulfon­yl)-4-(*substituted*)benzamides, gave 14 hits. These fourteen compounds along with the three title compounds, (I)–(III), are grouped into three series; **series 1**: *N*-(2-methyl­phenyl­sulfon­yl)benzamide, *N*-(2-methyl­phen­yl­sulfon­yl)-4-(*chloro/meth­yl/nitro/meth­oxy*)benzamides and (I), **series 2**: *N*-(2-chloro­phenyl­sulfon­yl)benzamide, *N*-(2-chloro­phenyl­sulfon­yl)-4-(*chloro/meth­yl/nitro/meth­oxy*)benzamides and (II), and **series 3**: *N*-(4-chloro­phenyl­sulfon­yl)benzamide, *N*-(4-chloro­phenyl­sulfon­yl)-4-(*chloro/meth­yl/nitro*)benzamides and (III).

**Series 1**: In series 1 (Table 4 ▸), the asymmetric units of three compounds, namely, *N*-(2-methyl­phenyl­sulfon­yl)benzamide (Suchetan *et al.*, 2010*d* ▸), *N*-(2-methyl­phenyl­sulfon­yl)-4-nitro­benzamide (Suchetan *et al.*, 2011*b* ▸) and *N*-(2-methyl­phenyl­sulfon­yl)-4-meth­oxy­benzamide (Sreenivasa *et al.*, 2014*a* ▸) contain one mol­ecule, while those of *N*-(2-methyl­phenyl­sulfon­yl)-4-chloro­benzamide (Suchetan *et al.*, 2010*e* ▸), *N*-(2-methyl­phenyl­sulfon­yl)-4-methyl­benzamide (Gowda *et al.*, 2010*a* ▸) and *N*-(2-methyl­phenyl­sulfon­yl)-4-fluoro­benzamide (I) contain two mol­ecules. In all of the compounds of series 1, the conformation of the *ortho*-methyl group on the benzene­sulfonyl ring is *syn* to the N—H bond in the central –C–SO_2-_-N–C(O)– segment. The values of the dihedral angle between the two aromatic rings in the mol­ecules of series 1 fall in the range 73.9 (1)– 89.4 (1)°, the smallest dihedral angle being in *N*-(2-methyl­phenyl­sulfon­yl)benzamide and the largest in *N*-(2-methyl­phenyl­sulfon­yl)-4-chloro­benzamide (Table 4 ▸). Comparison of the inter­molecular inter­actions displayed in the crystal structures of compounds in this series reveals that, except for the meth­oxy- and fluoro-substituted compounds, the crystal structures all display N—H⋯O(S) hydrogen bonds, while the meth­oxy- and fluoro-substituted compounds display other weak inter­actions of the type C—H⋯O, C—H⋯π_ar­yl_, π_ar­yl_–π_ar­yl_ in addition to the N—H⋯O(S) hydrogen bonds. However, except for compound (I), all the compounds display one-dimensional supra­molecular chains, whereas in (I), the supra­molecular architecture is three-dimensional.

**Series 2:** The asymmetric units of all of the compounds in series 2 (Table 5 ▸) contain one mol­ecule and the conformation of the *ortho*-chloro substituent on the benzene­sulfonyl ring is *syn* to the N—H bond in the central –C–SO_2_–N–C(O)– segment. The values of the dihedral angle between the two aromatic rings in the mol­ecules fall in the range 73.3 (1)–89.91 (10)°, which is almost the same as in series 1, the smallest being in *N*-(2-chloro­phenyl­sulfon­yl)benzamide (Gowda *et al.*, 2010*b* ▸) and the largest in *N*-(2-chloro­phenyl­sulfon­yl)-4-fluoro­benzamide (II) (Table 5 ▸). The crystal structures of *N*-(2-chloro­phenyl­sulfon­yl)-benzamide, *N*-(2-chloro­phenyl­sulfon­yl)-4-chloro­benzamide (Suchetan *et al.*, 2011*c* ▸) and *N*-(2-chloro­phenyl­sulfon­yl)-4-methyl­benzamide (Gowda *et al.*, 2010*c* ▸) display zero-dimensional architectures featuring inversion-related 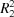
(8) dimers formed *via* N—H⋯O(S) hydrogen bonds, while strong N—H⋯O(S) hydrogen bonds in *N*-(2-chloro­phenyl­sulfon­yl)-4-nitro­benzamide (Suchetan *et al.*, 2011*d* ▸) lead to one-dimensional chains. Similar to that observed in series 1, the meth­oxy- and fluoro-substituted compounds in series 2 show diversity in their inter­molecular inter­actions. *N*-(2-chloro­phenyl­sulfon­yl)-4-meth­oxy­benzamide (Sreenivasa *et al.*, 2014*b* ▸) features structure-directing N—H⋯O(S) and C—H⋯O(S) hydrogen bonds and weak π_ar­yl_–π_ar­yl_ inter­actions, resulting in a two-dimensional structure. However, in *N*-(2-chloro­phenyl­sulfon­yl)-4-fluoro­benzamide (II), N—H⋯O(S) and C—H⋯O(S) hydrogen bonds (with no structure-directing characteristics) between mol­ecules form inversion-related dimers, and these dimers are inter­connected *via* C=O⋯π_ar­yl_ inter­actions, forming a one-dimensional architecture.

**Series 3**: In series 3, the parent compound *N*-(4-chloro­phenyl­sulfon­yl)benzamide (Suchetan *et al.*, 2010*a* ▸) crystallizes with two mol­ecules in the asymmetric unit, while *N*-(4-chloro­phenyl­sulfon­yl)-4-chloro­benzamide (Suchetan *et al.*, 2010*b* ▸), *N*-(4-chloro­phenyl­sulfon­yl)-4-methyl­benzamide (Suchetan *et al.*, 2010*c* ▸) and *N*-(4-chloro­phenyl­sulfon­yl)-4-nitro­benzamide (Suchetan *et al.*, 2011*a* ▸) crystallize with one mol­ecule, and *N*-(4-chloro­phenyl­sulfon­yl)-4-fluoro­benzamide (III) crystallizes with one mol­ecule and a mol­ecule of water in the asymmetric unit. The values of the dihedral angle between the two aromatic rings in the mol­ecules are in the range 62.8 (1)–89.5 (1)°, the smallest value being for *N*-(4-chloro­phenyl­sulfon­yl)benzamide and the largest for *N*-(4-chloro­phenyl­sulfon­yl)-4-methyl­benzamide (Table 6 ▸). Except for compound (III), the crystals of all of the compounds feature N—H⋯O(S) hydrogen bonds, either forming 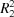
(8) inversion dimers (zero-dimensional structure) or one-dimensional chains. Once again, the fluoro-substituted compound (III) displays a variety of hydrogen bonds and weak inter­actions (Tables 3 ▸ and 6 ▸), leading to a three-dimensional architecture.

## Synthesis and crystallization   

Compounds (I)–(III) were prepared by refluxing a mixture of 4-fluoro­benzoic acid, the corresponding substituted benzene­sulfonamides and phospho­rousoxychloride for 3 h on a water bath. The resultant mixtures were cooled and poured into ice-cold water. The solids obtained were filtered, washed thoroughly with water and then dissolved in sodium bicarbonate solutions. The compounds were later re-precipitated by acidifying the filtered solutions with dilute HCl. They were filtered, dried and recrystallized. [Melting point (m.p.) of (I) = 410 K, (II) = 428 K and (III) = 456 K]. Prism-like, colourless single crystals of all three of the compounds were obtained from slow evaporation of the respective solutions of the compounds in methanol (with few drops of water).

## Refinement   

Crystal data, data collection and structure refinement details are summarized in Table 7 ▸. The H atoms of the NH groups in (I)–(III) were located in difference Fourier maps and freely refined. The H atoms of the water mol­ecule in (III) were located in a difference Fourier map and were refined with the bond length restraint O—H = 0.83 (3) Å. The other H atoms were positioned with idealized geometry using a riding model: C—H = 0.93–0.96 Å, with *U*_iso_ = 1.5*U*_eq_(C-meth­yl) and 1.2*U*_eq_(C) for other H atoms. In the final cycles of refinement, reflections (0 1 1), (0 0 2) and (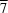 0 20) in (I), (0 0 2) in (II) and (2 0 0) in (III) were omitted due to large differences in *F* ^2^_obs_ and *F* ^2^_calc_.

## Supplementary Material

Crystal structure: contains datablock(s) I, II, III, global. DOI: 10.1107/S2056989016005089/su5287sup1.cif

CCDC references: 1470505, 1470504, 1470503

Additional supporting information:  crystallographic information; 3D view; checkCIF report

## Figures and Tables

**Figure 9:**
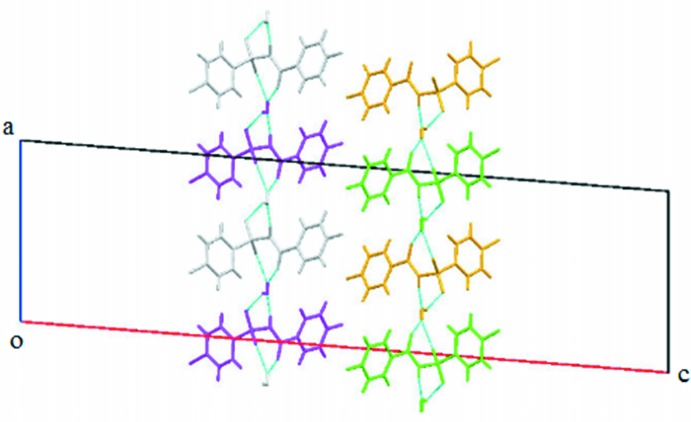
Crystal packing of (III) when viewed along the *b* axis; adjacent two-dimensional sheets are seen.

**Table 1:** Hydrogen-bond geometry (Å, °) for (I) *Cg* is the centroid of the fluoro­benzene ring of mol­ecule *B* of (I).

*D*—H⋯*A*	*D*—H	H⋯*A*	*D*⋯*A*	*D*—H⋯*A*
N1*A*—H1*A*⋯O1*B*	0.81 (3)	2.12 (3)	2.918 (2)	167 (2)
N1*B*—H1*B*⋯O1*A* ^i^	0.83 (3)	2.02 (3)	2.828 (3)	162 (3)
C6*A*—H6*A*⋯O3*B*	0.93	2.57	3.313 (3)	137
C10*B*—H10*B*⋯O1*B* ^ii^	0.93	2.59	3.376 (2)	143
C10*B*—H10*B*⋯O3*B* ^ii^	0.93	2.46	3.215 (2)	139
C4*B*—H4*B*⋯*Cg* ^iii^	0.93	2.72	3.646 (2)	173
C14*B*—H14*B*⋯O2*B*	0.96	2.45	3.058 (3)	121

Symmetry codes: (i) 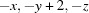; (ii) 
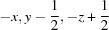
; (iii) 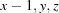.

**Table 2:** Hydrogen-bond geometry (Å, °) for (II)

*D*—H⋯*A*	*D*—H	H⋯*A*	*D*⋯*A*	*D*—H⋯*A*
N1—H1⋯O2^i^	0.82 (3)	2.16 (3)	2.968 (2)	172 (2)
C13—H13⋯O2^i^	0.93	2.40	3.194 (2)	144

Symmetry code: (i) 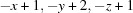.

**Table 3:** Hydrogen-bond geometry (Å, °) for (III)

*D*—H⋯*A*	*D*—H	H⋯*A*	*D*⋯*A*	*D*—H⋯*A*
N1—H1⋯O4	0.83 (3)	1.91 (3)	2.733 (3)	171 (2)
O4—H1*O*4⋯O1^i^	0.79 (3)	2.25 (3)	2.884 (2)	138 (3)
O4—H2*O*4⋯O2^ii^	0.82 (2)	2.29 (3)	2.955 (2)	139 (3)
O4—H2*O*4⋯O3^ii^	0.82 (2)	2.16 (3)	2.841 (2)	141 (3)
C5—H5⋯O1^iii^	0.93	2.54	3.124 (3)	121

Symmetry codes: (i) 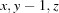; (ii) 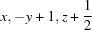; (iii) 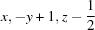.

**Table 4:** Comparison of various parameters (°) in the crystal structures of series 1: *N*-(2-methyl­phenyl­sulfon­yl)-*para*-substituted-aryl­amides

Parameters	H	Cl	CH_3_	NO_2_	OCH_3_	F
Crystal System	Ortho­rhom­bic	Triclinic	Triclinic	Monoclinic	Monoclinic	Monoclinic
*Z*′	1	2	2	1	1	2
Orientation of 2-CH_3_ group to the N—H bond	*syn*	*syn*, *syn*	*syn*, *syn*	*syn*	*syn*	*syn*, *syn*
Angle between aromatic rings	73.9 (1)	89.4 (1), 82.4 (1)	88.1 (1), 83.5 (1)	83.8 (2)	80.81 (1)	82.83 (11), 85.01 (10)
Inter­molecular inter­actions	N—H⋯O(S)	N—H⋯O(S)	N—H⋯O(S)	N—H⋯O(S)	N—H⋯O(S), C—H⋯O(S), π–π	N—H⋯O(S), C—H⋯O(S), C—H⋯π, π–π, S=O⋯π
Supra­molecular architecture	0D chains	0D	0D chains	0D chains	1D chains	3D

**Table 5:** Comparison of various parameters (°) in the crystal structures of series 2: *N*-(2-chloro­phenyl­sulfon­yl)-*para*-substituted-aryl­amides

Parameters	H	Cl	CH_3_	NO_2_	OCH_3_	F
Crystal System	Triclinic	Triclinic	Monoclinic	Monoclinic	Monoclinic	Monoclinic
*Z*′	1	1	1	1	1	1
Orientation of 2-Cl group to the N—H bond	*syn*	*syn*	*syn*	*syn*	*syn*	*syn*
Angle between aromatic rings	73.3 (1)	85.7 (1)	89.1 (2)	85.4 (1)	82.07 (1)	89.9 (1)
Inter­molecular inter­actions	N—H⋯O(S)	N—H⋯O(S)	N—H⋯O(S)	N—H⋯O(S)	N—H⋯O(S), C—H⋯O(S), π–p	N—H⋯O(S), C—H⋯O(S), C=O⋯π
Supra­molecular architecture	0D (ring motifs)	0D (ring motifs)	0D (ring motifs)	1D chains	2D	1D

**Table 6:** Comparison of various parameters (°) in the crystal structures of series 3: *N*-(4-chloro­phenyl­sulfon­yl)-*para*-substituted-aryl­amides

Parameters	H	Cl	CH_3_	NO_2_	F
Crystal System	Triclinic	Ortho­rhom­bic	Ortho­rhom­bic	Monoclinic	Monoclinic
*Z*′	2	1	1	1	1, H_2_O
Angle between aromatic rings	62.8 (1), 78.6 (1)	85.6 (1)	89.5 (1)	87.8 (1)	81.82 (11)
Inter­molecular inter­actions	N—H⋯O(S)	N—H⋯O(S)	N—H⋯O(C)	N—H⋯O(S)	N—H⋯O(*W*), O(*W*)—H⋯O(S), O(*W*)—H⋯O(C), C—H⋯O(S), C—Cl⋯π, C—F⋯π, S=O⋯π
Supra­molecular architecture	0D (ring motifs)	0D chains	0D (ring motifs)	D chains	3D

**Table 7:** Experimental details

	(I)	(II)	(III)
Crystal data			
Chemical formula	C_14_H_12_FNO_3_S	C_13_H_9_ClFNO_3_S	C_13_H_9_ClFNO_3_S·H_2_O
*M*_r_	293.31	313.72	331.74
Crystal system, space group	Monoclinic, *P*2_1_/*c*	Monoclinic, *P*2_1_/*c*	Monoclinic, *C*2/*c*
Temperature (K)	173	173	173
*a*, *b*, *c* (Å)	10.0259 (3), 12.4289 (3), 21.6241 (6)	7.9009 (2), 9.0775 (3), 18.4216 (5)	45.5989 (11), 4.8853 (1), 12.6517 (3)
β (°)	92.443 (1)	99.801 (1)	94.481 (1)
*V* (Å^3^)	2692.15 (13)	1301.92 (6)	2809.73 (11)
*Z*	8	4	8
Radiation type	Cu *K*α	Cu *K*α	Cu *K*α
μ (mm^−1^)	2.32	4.29	4.06
Crystal size (mm)	0.28 × 0.26 × 0.21	0.30 × 0.27 × 0.23	0.28 × 0.25 × 0.23

Data collection			
Diffractometer	Bruker APEXII	Bruker APEXII	Bruker APEXII
Absorption correction	Multi-scan (*SADABS*; Bruker, 2009 ▸)	Multi-scan (*SADABS*; Bruker, 2009 ▸)	Multi-scan (*SADABS*; Bruker, 2009 ▸)
*T*_min_, *T*_max_	0.548, 0.614	0.317, 0.373	0.369, 0.393
No. of measured, independent and observed [*I* > 2σ(*I*)] reflections	26667, 4404, 4165	10032, 2124, 2074	11176, 2320, 2030
*R*_int_	0.042	0.042	0.054
(sin θ/λ)_max_ (Å^−1^)	0.587	0.585	0.584

Refinement			
*R*[*F* ^2^ > 2σ(*F* ^2^)], *wR*(*F* ^2^), *S*	0.040, 0.121, 1.00	0.041, 0.124, 0.97	0.039, 0.118, 0.94
No. of reflections	4404	2124	2320
No. of parameters	371	185	205
No. of restraints	0	0	2
H-atom treatment	H atoms treated by a mixture of independent and constrained refinement	H atoms treated by a mixture of independent and constrained refinement	H atoms treated by a mixture of independent and constrained refinement
Δρ_max_, Δρ_min_ (e Å^−3^)	0.36, −0.46	0.37, −0.50	0.34, −0.35

Computer programs: *APEX2*, *SAINT-Plus* and *XPREP* (Bruker, 2009 ▸), *SHELXS97* and *SHELXL97* (Sheldrick, 2008 ▸) and *Mercury* (Macrae *et al.*, 2008 ▸).

## supplementary crystallographic information

### Crystal data

**Table:**

C_13_H_9_ClFNO_3_S·H_2_O	Prism
*M**_r_* = 331.74	*D*_x_ = 1.568 Mg m^−^^3^
Monoclinic, *C*2/*c*	Melting point: 456 K
Hall symbol: -C 2yc	Cu *K*α radiation, λ = 1.54178 Å
*a* = 45.5989 (11) Å	Cell parameters from 163 reflections
*b* = 4.8853 (1) Å	θ = 5.8–64.3°
*c* = 12.6517 (3) Å	µ = 4.06 mm^−^^1^
β = 94.481 (1)°	*T* = 173 K
*V* = 2809.73 (11) Å^3^	Prism, colourless
*Z* = 8	0.28 × 0.25 × 0.23 mm
*F*(000) = 1360	

### Data collection

**Table:**

Bruker APEXII diffractometer	2030 reflections with *I* > 2σ(*I*)
Radiation source: fine-focus sealed tube	*R*_int_ = 0.054
Graphite monochromator	θ_max_ = 64.3°, θ_min_ = 5.8°
phi and φ scans	*h* = −52→51
Absorption correction: multi-scan (*SADABS*; Bruker, 2009)	*k* = −5→5
*T*_min_ = 0.369, *T*_max_ = 0.393	*l* = −14→14
11176 measured reflections	1 standard reflections every 1 reflections
2320 independent reflections	intensity decay: 0.1%

### Refinement

**Table:**

Refinement on *F*^2^	Primary atom site location: structure-invariant direct methods
Least-squares matrix: full	Secondary atom site location: difference Fourier map
*R*[*F*^2^ > 2σ(*F*^2^)] = 0.039	Hydrogen site location: inferred from neighbouring sites
*wR*(*F*^2^) = 0.118	H atoms treated by a mixture of independent and constrained refinement
*S* = 0.94	*w* = 1/[σ^2^(*F*_o_^2^) + (0.0912*P*)^2^ + 2.087*P*] where *P* = (*F*_o_^2^ + 2*F*_c_^2^)/3
2320 reflections	(Δ/σ)_max_ = 0.001
205 parameters	Δρ_max_ = 0.34 e Å^−^^3^
2 restraints	Δρ_min_ = −0.35 e Å^−^^3^

### Special details

**Table:**

Geometry. All s.u.'s (except the s.u. in the dihedral angle between two l.s. planes) are estimated using the full covariance matrix. The cell s.u.'s are taken into account individually in the estimation of s.u.'s in distances, angles and torsion angles; correlations between s.u.'s in cell parameters are only used when they are defined by crystal symmetry. An approximate (isotropic) treatment of cell s.u.'s is used for estimating s.u.'s involving l.s. planes.
Refinement. Refinement of *F*^2^ against ALL reflections. The weighted *R*-factor *wR* and goodness of fit *S* are based on *F*^2^, conventional *R*-factors *R* are based on *F*, with *F* set to zero for negative *F*^2^. The threshold expression of *F*^2^ > 2σ(*F*^2^) is used only for calculating *R*-factors(gt) *etc*. and is not relevant to the choice of reflections for refinement. *R*-factors based on *F*^2^ are statistically about twice as large as those based on *F*, and *R*- factors based on ALL data will be even larger.

### Fractional atomic coordinates and isotropic or equivalent isotropic displacement parameters (Å^2^)

**Table:**

	*x*	*y*	*z*	*U*_iso_*/*U*_eq_	
S1	0.356285 (11)	0.64551 (10)	0.55127 (4)	0.0147 (2)	
Cl1	0.261887 (12)	−0.14924 (12)	0.34233 (4)	0.0273 (2)	
O2	0.36322 (3)	0.8548 (3)	0.47847 (11)	0.0176 (4)	
O1	0.34790 (3)	0.7192 (3)	0.65445 (11)	0.0195 (4)	
F1	0.49577 (3)	−0.3420 (3)	0.62191 (12)	0.0384 (4)	
O3	0.39675 (3)	0.4091 (3)	0.40288 (11)	0.0207 (4)	
O4	0.38058 (4)	0.1618 (4)	0.75835 (13)	0.0290 (4)	
N1	0.38535 (4)	0.4484 (4)	0.57495 (14)	0.0161 (4)	
C7	0.40210 (5)	0.3508 (4)	0.49595 (16)	0.0165 (5)	
C8	0.42713 (5)	0.1710 (4)	0.53341 (17)	0.0174 (5)	
C3	0.28843 (5)	0.1252 (5)	0.50950 (18)	0.0216 (5)	
H3	0.2753	0.0371	0.5507	0.026*	
C13	0.43853 (5)	−0.0018 (5)	0.45932 (17)	0.0218 (5)	
H13	0.4305	−0.0004	0.3895	0.026*	
C2	0.30931 (5)	0.3027 (5)	0.55508 (17)	0.0206 (5)	
H2	0.3105	0.3346	0.6278	0.025*	
C1	0.32848 (4)	0.4331 (4)	0.49134 (16)	0.0155 (5)	
C6	0.32692 (5)	0.3910 (5)	0.38239 (16)	0.0174 (5)	
H6	0.3397	0.4821	0.3406	0.021*	
C5	0.30619 (5)	0.2126 (5)	0.33712 (16)	0.0189 (5)	
H5	0.3049	0.1814	0.2644	0.023*	
C4	0.28742 (5)	0.0806 (5)	0.40070 (17)	0.0190 (5)	
C9	0.43954 (5)	0.1702 (5)	0.63795 (18)	0.0241 (5)	
H9	0.4321	0.2858	0.6878	0.029*	
C10	0.46276 (5)	−0.0009 (6)	0.66765 (19)	0.0295 (6)	
H10	0.4712	−0.0013	0.7369	0.035*	
C12	0.46171 (5)	−0.1755 (5)	0.48843 (19)	0.0265 (6)	
H12	0.4694	−0.2917	0.4393	0.032*	
C11	0.47306 (5)	−0.1710 (5)	0.5923 (2)	0.0264 (6)	
H1O4	0.3743 (8)	0.011 (6)	0.761 (3)	0.067 (12)*	
H1	0.3854 (6)	0.373 (6)	0.634 (2)	0.037 (8)*	
H2O4	0.3812 (7)	0.229 (6)	0.8178 (19)	0.039 (8)*	

### Atomic displacement parameters (Å^2^)

**Table:**

	*U*^11^	*U*^22^	*U*^33^	*U*^12^	*U*^13^	*U*^23^
S1	0.0164 (3)	0.0130 (3)	0.0148 (3)	0.0011 (2)	0.0022 (2)	−0.00059 (18)
Cl1	0.0245 (4)	0.0239 (4)	0.0326 (4)	−0.0091 (2)	−0.0040 (3)	0.0024 (2)
O2	0.0191 (8)	0.0126 (8)	0.0213 (8)	0.0000 (6)	0.0024 (6)	0.0004 (6)
O1	0.0221 (8)	0.0194 (8)	0.0173 (7)	0.0012 (7)	0.0040 (6)	−0.0034 (6)
F1	0.0277 (8)	0.0398 (10)	0.0466 (9)	0.0179 (7)	−0.0037 (7)	0.0018 (7)
O3	0.0240 (8)	0.0209 (8)	0.0173 (8)	0.0031 (7)	0.0034 (6)	0.0016 (6)
O4	0.0504 (12)	0.0206 (11)	0.0165 (8)	−0.0034 (8)	0.0067 (8)	−0.0021 (7)
N1	0.0180 (10)	0.0154 (10)	0.0149 (9)	0.0003 (8)	0.0004 (7)	0.0021 (7)
C7	0.0165 (11)	0.0135 (11)	0.0195 (11)	−0.0041 (8)	0.0010 (8)	−0.0018 (8)
C8	0.0145 (11)	0.0155 (11)	0.0225 (11)	−0.0025 (8)	0.0030 (8)	0.0011 (8)
C3	0.0181 (11)	0.0233 (13)	0.0240 (12)	−0.0016 (9)	0.0048 (9)	0.0060 (9)
C13	0.0210 (11)	0.0217 (13)	0.0227 (11)	0.0006 (10)	0.0017 (9)	−0.0018 (9)
C2	0.0211 (12)	0.0232 (13)	0.0175 (11)	0.0012 (10)	0.0022 (9)	0.0013 (9)
C1	0.0156 (11)	0.0114 (11)	0.0195 (10)	0.0029 (9)	0.0014 (8)	0.0003 (8)
C6	0.0159 (11)	0.0176 (12)	0.0191 (10)	0.0016 (9)	0.0031 (8)	0.0031 (9)
C5	0.0183 (11)	0.0213 (12)	0.0168 (10)	0.0010 (9)	0.0001 (8)	−0.0011 (9)
C4	0.0158 (11)	0.0133 (11)	0.0273 (11)	−0.0003 (9)	−0.0024 (8)	0.0010 (9)
C9	0.0224 (12)	0.0268 (14)	0.0230 (11)	0.0033 (10)	0.0005 (9)	−0.0032 (9)
C10	0.0225 (12)	0.0397 (16)	0.0255 (12)	0.0043 (11)	−0.0040 (9)	0.0016 (11)
C12	0.0227 (12)	0.0236 (14)	0.0335 (13)	0.0022 (10)	0.0045 (10)	−0.0069 (10)
C11	0.0163 (12)	0.0251 (14)	0.0374 (13)	0.0061 (10)	−0.0016 (10)	0.0050 (10)

### Geometric parameters (Å, º)

**Table:**

S1—O2	1.4279 (15)	C3—H3	0.9300
S1—O1	1.4346 (14)	C13—C12	1.382 (3)
S1—N1	1.6466 (18)	C13—H13	0.9300
S1—C1	1.763 (2)	C2—C1	1.389 (3)
Cl1—C4	1.740 (2)	C2—H2	0.9300
F1—C11	1.360 (3)	C1—C6	1.390 (3)
O3—C7	1.217 (3)	C6—C5	1.377 (3)
O4—H1O4	0.79 (3)	C6—H6	0.9300
O4—H2O4	0.82 (2)	C5—C4	1.380 (3)
N1—C7	1.389 (3)	C5—H5	0.9300
N1—H1	0.84 (3)	C9—C10	1.378 (3)
C7—C8	1.488 (3)	C9—H9	0.9300
C8—C13	1.392 (3)	C10—C11	1.375 (4)
C8—C9	1.397 (3)	C10—H10	0.9300
C3—C2	1.380 (3)	C12—C11	1.374 (3)
C3—C4	1.391 (3)	C12—H12	0.9300
			
O2—S1—O1	119.70 (9)	C2—C1—C6	121.5 (2)
O2—S1—N1	108.70 (9)	C2—C1—S1	118.95 (16)
O1—S1—N1	104.43 (9)	C6—C1—S1	119.53 (16)
O2—S1—C1	109.39 (9)	C5—C6—C1	119.1 (2)
O1—S1—C1	107.77 (9)	C5—C6—H6	120.4
N1—S1—C1	105.99 (10)	C1—C6—H6	120.4
H1O4—O4—H2O4	109 (3)	C6—C5—C4	119.3 (2)
C7—N1—S1	123.36 (15)	C6—C5—H5	120.3
C7—N1—H1	122 (2)	C4—C5—H5	120.3
S1—N1—H1	112 (2)	C5—C4—C3	122.0 (2)
O3—C7—N1	122.4 (2)	C5—C4—Cl1	118.63 (17)
O3—C7—C8	122.48 (19)	C3—C4—Cl1	119.37 (17)
N1—C7—C8	115.13 (18)	C10—C9—C8	120.4 (2)
C13—C8—C9	119.4 (2)	C10—C9—H9	119.8
C13—C8—C7	117.45 (19)	C8—C9—H9	119.8
C9—C8—C7	123.2 (2)	C9—C10—C11	118.3 (2)
C2—C3—C4	118.7 (2)	C9—C10—H10	120.8
C2—C3—H3	120.6	C11—C10—H10	120.8
C4—C3—H3	120.6	C11—C12—C13	117.9 (2)
C12—C13—C8	120.7 (2)	C11—C12—H12	121.0
C12—C13—H13	119.6	C13—C12—H12	121.0
C8—C13—H13	119.6	F1—C11—C12	118.4 (2)
C3—C2—C1	119.4 (2)	F1—C11—C10	118.3 (2)
C3—C2—H2	120.3	C12—C11—C10	123.3 (2)
C1—C2—H2	120.3		
			
O2—S1—N1—C7	−45.29 (19)	O1—S1—C1—C6	163.03 (17)
O1—S1—N1—C7	−174.12 (17)	N1—S1—C1—C6	−85.63 (19)
C1—S1—N1—C7	72.20 (19)	C2—C1—C6—C5	−1.0 (3)
S1—N1—C7—O3	1.5 (3)	S1—C1—C6—C5	176.33 (16)
S1—N1—C7—C8	−178.80 (14)	C1—C6—C5—C4	0.2 (3)
O3—C7—C8—C13	−20.8 (3)	C6—C5—C4—C3	1.0 (3)
N1—C7—C8—C13	159.45 (19)	C6—C5—C4—Cl1	−178.71 (16)
O3—C7—C8—C9	158.7 (2)	C2—C3—C4—C5	−1.4 (3)
N1—C7—C8—C9	−21.0 (3)	C2—C3—C4—Cl1	178.32 (18)
C9—C8—C13—C12	0.7 (3)	C13—C8—C9—C10	−0.3 (3)
C7—C8—C13—C12	−179.7 (2)	C7—C8—C9—C10	−179.8 (2)
C4—C3—C2—C1	0.5 (3)	C8—C9—C10—C11	−0.6 (4)
C3—C2—C1—C6	0.7 (3)	C8—C13—C12—C11	−0.2 (3)
C3—C2—C1—S1	−176.74 (17)	C13—C12—C11—F1	179.7 (2)
O2—S1—C1—C2	−151.16 (17)	C13—C12—C11—C10	−0.8 (4)
O1—S1—C1—C2	−19.5 (2)	C9—C10—C11—F1	−179.3 (2)
N1—S1—C1—C2	91.82 (19)	C9—C10—C11—C12	1.2 (4)
O2—S1—C1—C6	31.4 (2)		

### Hydrogen-bond geometry (Å, º)

**Table:**

*D*—H···*A*	*D*—H	H···*A*	*D*···*A*	*D*—H···*A*
N1—H1···O4	0.83 (3)	1.91 (3)	2.733 (3)	171 (2)
O4—H1*O*4···O1^i^	0.79 (3)	2.25 (3)	2.884 (2)	138 (3)
O4—H2*O*4···O2^ii^	0.82 (2)	2.29 (3)	2.955 (2)	139 (3)
O4—H2*O*4···O3^ii^	0.82 (2)	2.16 (3)	2.841 (2)	141 (3)
C5—H5···O1^iii^	0.93	2.54	3.124 (3)	121

Symmetry codes: (i) *x*, *y*−1, *z*; (ii) *x*, −*y*+1, *z*+1/2; (iii) *x*, −*y*+1, *z*−1/2.
